# Supplementary material for: Predicting visual function by interpreting a neuronal wiring diagram
Source: Nature. 2024 Oct 2;634(8032):113–23. doi: 10.1038/s41586-024-07953-5 (PMC11446822; doi:10.1038/s41586-024-07953-5)
Supplement: Supplementary file 6 — CRF and ERF predictions for individual TmY4 and TmY9 cells. Analogous to Supplementary Data 3, but for TmY target types. Shown are the top four monosynaptic pathways, the strongest pathway passing through each of the top ten intermediary types (ranking from Extended Data Fig. 7), and the trisynaptic pathway Tm1–TmY–Dm3–TmY (see the section entitled Prediction of spatial normalization). [file 41586_2024_7953_MOESM6_ESM.zip › DataS4/TmY9qΓèÑ/720575940631135786.pdf]

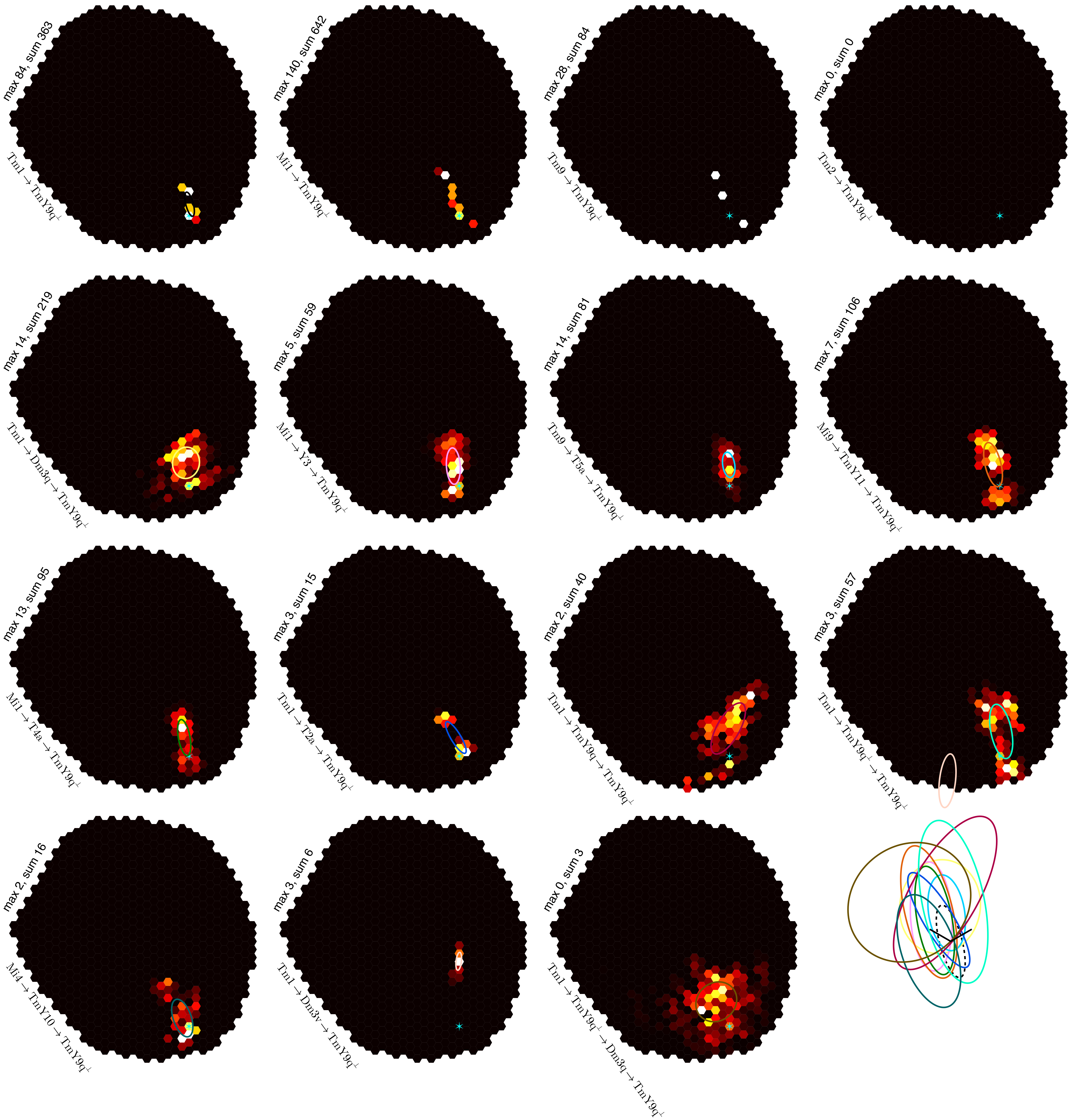

max 84, sum 363
Tm1 → TmY9q⊥
max 140, sum 642
Mi1 → TmY9q⊥
max 28, sum 84
Tm9 → TmY9q⊥
max 0, sum 0
Tm2 → TmY9q⊥
max 14, sum 219
Tm1 → Dm3q → TmY9q⊥
max 5, sum 59
Mi1 → Y3 → TmY9q⊥
max 14, sum 81
Tm9 → T5a → TmY9q⊥
max 7, sum 106
Mi9 → TmY11 → TmY9q⊥
max 13, sum 95
Mi1 → T4a → TmY9q⊥
max 3, sum 15
Tm1 → T2a → TmY9q⊥
max 2, sum 40
Tm1 → TmY9q → TmY9q⊥
max 3, sum 57
Tm1 → TmY9q⊥ → TmY9q⊥
max 2, sum 16
Mi4 → TmY10 → TmY9q⊥
max 3, sum 6
Tm1 → Dm3v → TmY9q⊥
max 0, sum 3
Tm1 → TmY9q⊥ → Dm3q → TmY9q⊥
